# Expression and therapeutic potential of TROP2 in cisplatin-resistant germ cell tumors

**DOI:** 10.1007/s00432-025-06325-4

**Published:** 2025-10-07

**Authors:** Laurenz Sperber, Melanie von Brandenstein, Carolina Kessler, Julian Heidenreich, Enno Storz, David Pfister, Pia Paffenholz, Yuri Tolkach, Marit Bernhardt, Ralph Wirtz, Markus Eckstein, Axel Heidenreich, Richard Weiten

**Affiliations:** 1 Department of Urology, Uro-Oncology, Robot-Assisted, and Specialized Urologic Surgery, University Hospital Cologne, Kerpener Str. 62, 50937 Cologne, Germany; 2 Institute of Pathology, University Hospital Cologne, Cologne, Germany; 3 Institute of Pathology, University Hospital Bonn, Bonn, Germany; 4 STRATIFYER Molecular Pathology GmbH, Cologne, Germany; 5 Institute of Pathology, University Hospital Erlangen, Friedrich-Alexander-Universität Erlangen-Nürnberg, Erlangen, Germany; 6 Department of Urology, Medical University Vienna, Vienna, Austria

**Keywords:** Antibody–drug conjugate, Choriocarcinoma, Cisplatin resistance, Germ cell tumors, TROP2

## Abstract

**Background and Objectives:**

Cisplatin-based chemotherapy remains the standard of care for metastatic germ cell tumors (GCTs), but patients with cisplatin-refractory disease have limited effective options and poor outcomes. There is a clear unmet need for new therapies in this setting. Antibody–drug conjugates (ADCs) have emerged as a promising class of targeted agents for chemoresistant solid tumors. This study aimed to evaluate the expression of TROP2 in cisplatin-resistant GCT metastases [MET(-R)] and to assess the cytotoxic efficacy of the anti-TROP2 ADC sacituzumab govitecan (SG) in GCT cell lines.

**Methods:**

TROP2 mRNA and protein expression levels were analyzed in 31 post-chemotherapy viable MET(-R) specimens, including embryonal carcinoma (EC), choriocarcinoma (CC), yolk sac tumor (YST), and teratoma (TER), using quantitative reverse transcription polymerase chain reaction and immunohistochemistry with H-score evaluation. In vitro analyses included Western blotting and cell viability assays to assess TROP2 protein expression and SG-mediated cytotoxicity in GCT cell lines.

**Results:**

*TROP2* mRNA expression was significantly higher in post-chemotherapy CC- and TER-MET(-R) compared to YST-MET(-R) (*p* < 0.01). Immunohistochemistry showed moderate to strong membranous TROP2 expression in CC-MET(-R) [with H-score ≥ 100, median H-score 280 (interquartile range, IQR 225.0–298.0)] and TER-MET(-R) [median H-score 255 (IQR 200.0–265.0)]. Conversely, EC- and YST-MET(-R) showed absent or weak TROP2 expression [EC median H-score 22.5 (IQR 5.0–73.8); YST median H-score 0 (IQR 0–12.5)]. In vitro, SG induces dose-dependent cytotoxicity in TROP2-positive GCT cells, including cisplatin-resistant subclones (*p* < 0.01).

**Conclusion:**

In summary, TROP2 is variably expressed in cisplatin-resistant metastatic GCTs, with high expression most often seen in CC. SG shows strong cytotoxicity in TROP2-positive, chemotherapy-resistant GCT cells; however, its clinical effectiveness still needs to be confirmed.

**Supplementary Information:**

The online version contains supplementary material available at 10.1007/s00432-025-06325-4.

## Introduction

The classification of germ cell tumors (GCTs) into seminomas and non-seminomas is important because these subtypes differ in their biology, treatment response, and prognosis (Cheng et al. 2018; Singla et al. 2025). Seminomas are generally more responsive to both chemotherapy and radiation, while non-seminomas, which include embryonal carcinoma (EC), choriocarcinoma (CC), yolk sac tumor (YST), and teratoma (TER), are more diverse and can be less responsive, especially in cases of teratoma and cisplatin resistance (Cheng et al. 2018; Singla et al. 2025).}

Epidemiology shows that GCTs are the most common malignancy in males aged 15–40 years and have a rising incidence. This emphasizes the need for effective salvage therapies, as a significant minority of patients with metastatic disease develop cisplatin-resistant GCTs, resulting in poor outcomes (Cheng et al. 2018; Singla et al. 2025). The high cure rates with first-line cisplatin-based regimens starkly contrast with the limited effectiveness of current options for refractory disease, which include salvage chemotherapy and, in some cases, high-dose chemotherapy with autologous stem cell transplant (Lorch et al. 2010; Travis et al. 2024).

This context drives the development of innovative strategies, such as antibody–drug conjugates (ADCs), which have become a promising new class of targeted agents for chemoresistant solid tumors because of their ability to deliver cytotoxic payloads directly to tumor cells that express specific antigens (Evmorfopoulos et al. 2024; Udvorková et al. 2024).

Trophoblast cell surface antigen 2 (TROP2) is a membrane glycoprotein frequently overexpressed in various epithelial tumors and serves as the target for the ADC sacituzumab govitecan (SG) (Goldenberg et al. 2018). SG has demonstrated significant clinical efficacy in heavily pretreated metastatic triple-negative breast cancer (Bardia et al. 2021a), and its mechanism—targeting TROP2 with a topoisomerase I inhibitor payload (SN-38)—is relevant for tumors that express TROP2. Preclinical data indicate that TROP2 is expressed in some GCTs, providing a rationale for investigating SG in this context (Pavone et al. 2021; Tekin et al. 2025).

Therefore, we aimed to evaluate TROP2 expression levels in metastases of patients with cisplatin-resistant GCTs [MET(-R)] and to assess the cytotoxic effectiveness of SG in GCT cell lines, including cisplatin-resistant subclones.

## Materials and methods

### Patient cohort

We conducted a retrospective single-center analysis of 31 patients with metastatic GCTs who experienced disease progression after at least two lines of platinum-based chemotherapy, including high-dose chemotherapy (HDCT). The cohort, treated between 2012 and 2023 at the University Hospital of Cologne, included cases of post-chemotherapy embryonal carcinoma [EC-MET(-R), n = 4], choriocarcinoma [CC-MET(-R), n = 4], yolk sac tumor [YST-MET(-R), n = 14], and teratoma [TER-MET(-R), n = 9]. Seminoma cases were excluded because they typically exhibit negligible expression of TROP2 (Dum et al. 2022; Tekin et al. 2025).

The clinicopathological features of all patients are detailed in Supplementary Table S1. Histopathological classification was conducted following the 8th edition of the TNM classification of malignant tumors (Bertero et al. 2018) and the 5th edition of the WHO classification for urogenital tumors (Moch et al. 2022).

The study received approval from the Ethics Committee of the Medical Faculty at the University of Cologne (approval number 23-1178) and was conducted in accordance with the principles of the Declaration of Helsinki. Written informed consent was obtained from all patients before their participation.

### RNA isolation and quantitative real-time PCR (qRT-PCR)

Total RNA was extracted from microdissected formalin-fixed paraffin-embedded (FFPE) tissue sections using a validated bead-based extraction method (XTRACT kit, STRATIFYER Molecular Pathology GmbH, Cologne, Germany) following established laboratory protocols. Microdissection was performed to precisely isolate tumor tissue under microscopic guidance, reducing contamination from non-tumor components such as stroma, blood vessels, or inflammatory cells, and ensuring high tumor purity for molecular analysis. TROP2 mRNA levels were measured by qRT-PCR using a validated TaqMan gene expression assay (STRATIFYER Molecular Pathology GmbH, Cologne, Germany). Expression levels were normalized to the housekeeping gene CALM2, as previously described.

### Immunohistochemistry

Membranous TROP2 protein expression was assessed using immunohistochemistry (IHC) with a BOND-MAX autostainer (Leica Biosystems, Wetzlar, Germany) according to an accredited staining protocol in a routine IHC lab. The primary antibody used was a monoclonal anti-TROP2 antibody (#ab214488, Abcam, Cambridge, UK) at a 1:1000 dilution, incubated for 32 min at 37 °C. For quality assurance, each staining batch included positive controls (e.g., placental trophoblast and urothelial carcinoma tissue with known strong membranous TROP2 expression) and negative controls (omission of the primary antibody) to verify assay sensitivity, specificity, and technical consistency.

Three independent board-certified pathologists (Y. Tolkach, M. Bernhardt, and M. Eckstein) evaluated membranous TROP2 expression using the H-score method. Staining results were categorized as negative (H-score 0–14), weak (H-score 15–99), moderate (H-score 100–199), or strong (H-score 200–300), based on established criteria (Klümper et al. 2022; Weiten et al. 2024).

### Cell lines and culture conditions

Human GCT cell lines, including embryonal carcinoma (NCCIT, 2102EP), choriocarcinoma (JAR, JEG-3, BeWo), and EC-YST intermediate (1411H), as well as their cisplatin-resistant subclones (-R), were generously provided by Prof. D. Nettersheim. Cells were maintained in RPMI-1640 (#P04-16500, PAN-Biotech GmbH, Aidenbach, Germany), DMEM (#P04-03550, PAN-Biotech GmbH, Aidenbach, Germany), or Ham´s F-12 (#P04-14500, PAN-Biotech GmbH, Aidenbach, Germany) medium supplemented with 10% fetal bovine serum and 0,8% streptomycin-penicillin antibiotics (10.000 units/ml Penicillin and 10.000 µg/ml Streptomycin; #15,140–122, Thermo Fisher Scientific, Darmstadt, Germany), at 37 °C in a humidified atmosphere containing 5% CO₂.

### Western blot analysis

Cells were lysed in RIPA buffer supplemented with protease inhibitors. Protein concentration was determined using the BCA Protein Assay Kit (#23,225, Thermo Fisher Scientific, Darmstadt, Germany). Equal amounts of protein were denatured, separated by SDS-PAGE, and transferred to nitrocellulose membranes (#GE10600002, Amersham Protran Premium Western Blotting Membrane, Merck, Darmstadt, Germany). Membranes were blocked with 5% non-fat milk in TBST (50 mM Tris, 150 mM NaCl, 0.05% Tween 20, pH 7.5) for 1 h and incubated overnight at 4 °C with primary antibodies against TROP2 (#ab214488, abcam, Cambridge, UK, dilution 1:2000) and GAPDH (#sc-47724, Santa Cruz Biotechnology, Dallas, Texas, USA, dilution 1:1000). HRP-conjugated secondary antibodies were applied for 1 h. Signal detection was performed using a ChemoStar ECL Imager (INTAS, Göttingen, Germany).

### Cell viability assay

The cytotoxic activity of sacituzumab govitecan (SG) was assessed using a crystal violet assay (n = 3 independent experiments). Cells (1 × 10^^4^ per well) were seeded into 48-well plates and treated with increasing concentrations of SG (0–50 µg/mL) for 72 h. After treatment, cells were fixed and stained with 0.05% crystal violet in 0.1% acetic acid. Absorbance was measured at 570 nm using a VICTOR Nivo UV–Vis spectrometer (PerkinElmer, Rodgau, Germany). Cell viability was expressed as a relative absorbance compared to untreated controls.

### Statistical analysis

Statistical analyses were conducted using SPSS (Version 28.0.1.1, IBM, https://www.ibm.com/de-de/analytics/spss-statistics-software) and GraphPad Prism (Version 9.4.0, GraphPad, http://www.graphpad.com/). Group comparisons were performed with either the Kruskal–Wallis test or parametric t-tests, as appropriate. All p-values were two-sided, with statistical significance set at *p* < 0.05.

## Results

### TROP2 mRNA and protein expression in cisplatin-resistant GCT metastases

**Fig. 1:**
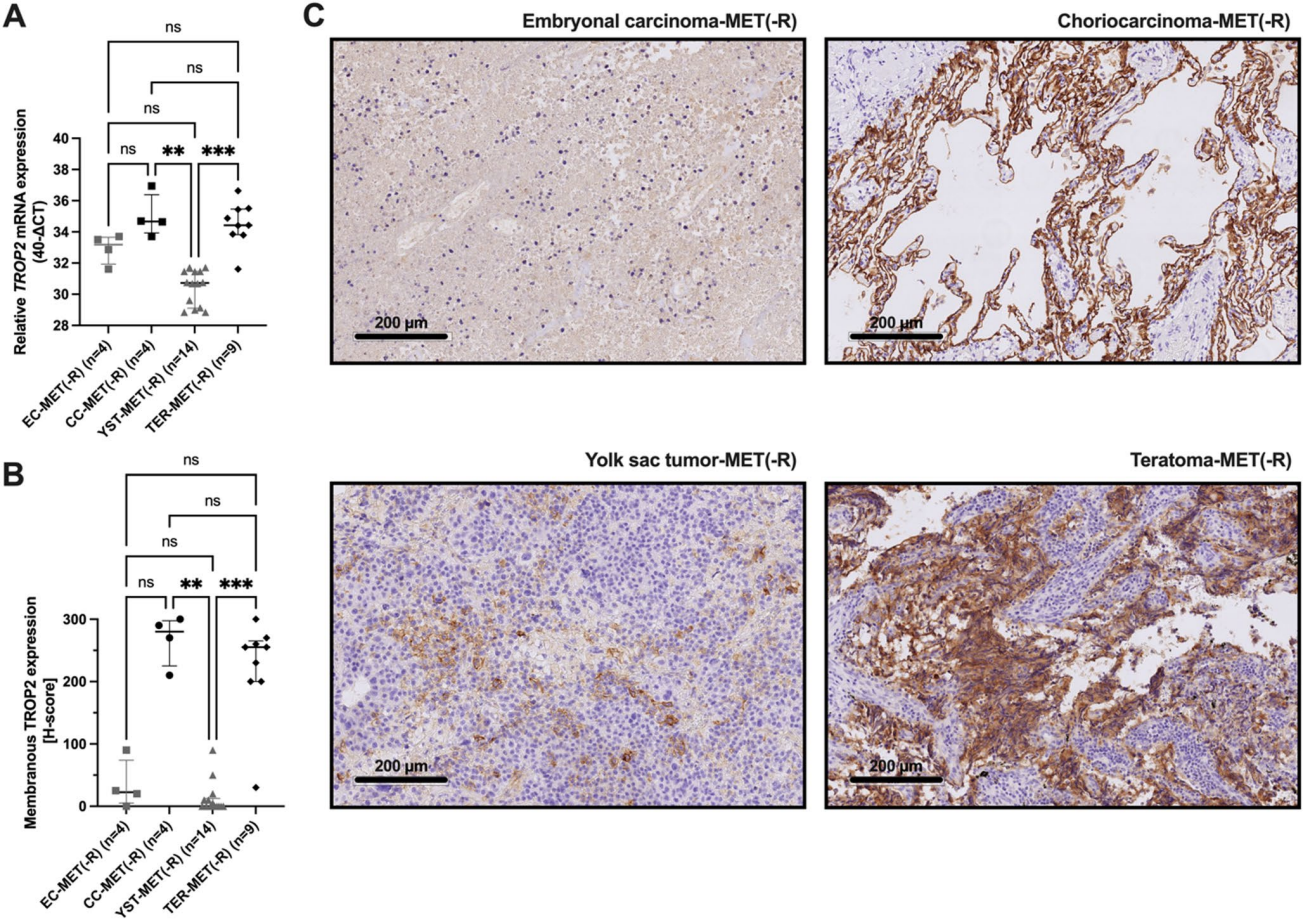
TROP2 mRNA and protein expression in cisplatin-resistant germ cell tumor (GCT) metastases. **A** Significantly increased TROP2 mRNA levels in post-chemotherapy choriocarcinoma [CC-MET(-R)] and teratoma metastases [TER-MET(-R)] compared to yolk sac tumor metastases [YST-MET(-R)]. **B** Moderate to strong membranous TROP2 protein expression in CC- [median H-score 280 (interquartile range, IQR 225–298)] and TER-MET(-R) [median H-score 255 (IQR 200–265)], whereas TROP2 expression was absent or weak in EC- and YST-MET(-R). **C** Representative immunohistochemical staining shows moderate to strong membranous TROP2 expression in CC-MET(-R) and TER-MET(-R), while weak or no expression was observed in EC-MET(-R) and YST-MET(-R) (200 × magnification). Kruskal–Wallis test, **p < 0.01, ***p < 0.001

In patients with cisplatin-resistant GCT metastases after chemotherapy, *TROP2* mRNA levels are significantly higher in choriocarcinoma metastases [CC-MET(-R)] and teratoma metastases [TER-MET(-R)] compared to yolk sac tumor metastases [YST-MET(-R)] (Kruskal–Wallis test, *p* < 0.01), while there is no statistically significant difference in mRNA expression among choriocarcinoma, teratoma, and embryonal carcinoma metastases [EC-MET(-R)] (Fig. 1A).

Immunohistochemically, moderate to strong membranous TROP2 protein expression is observed in CC- and TER-MET(-R) (with high median H-scores: 280 for CC, 255 for TER, and interquartile ranges, IQR 225–298 for CC, 200–265 for TER). In contrast, EC-MET(-R) shows absent or weak TROP2 expression (median H-score 22.5, IQR 5.0–73.8), and YST-MET(-R) displays no detectable TROP2 expression (median H-score 0, IQR 0–12.5) (Fig. 1B). These findings are consistent across both mRNA and protein analyses, indicating that TROP2 is most strongly expressed in CC and TER metastases but is mostly absent in YST metastases and variably expressed in EC metastases.

Representative immunohistochemical images in Fig. 1C show strong membranous TROP2 staining in CC-MET(-R) and TER-MET(-R), indicated by intense, continuous brown staining along the tumor cell membranes, reflecting high TROP2 protein levels. Conversely, EC and YST metastases display little or no membranous staining, with EC showing only weak or focal signals, and YST generally lacking detectable TROP2 expression. This pattern aligns with the quantitative H-score data, where CC-MET(-R) and TER-MET(-R) have high median H-scores, while EC and YST demonstrate low or undetectable scores, confirming that TROP2 expression is specific to certain cisplatin-resistant GCT subtypes.

### Cytotoxic efficacy of sacituzumab govitecan in GCT cell lines

**Fig. 2:**
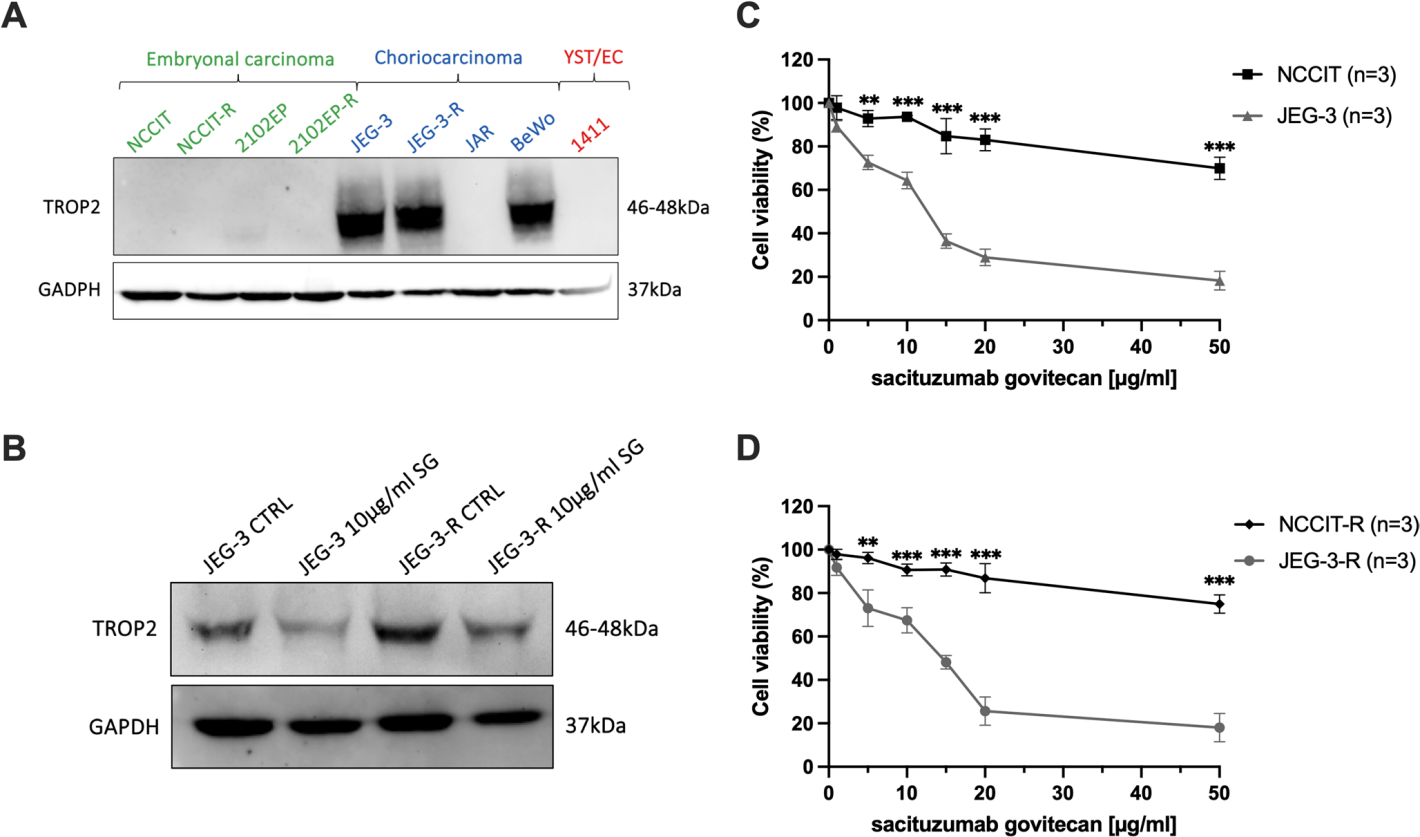
Cytotoxic effectiveness of sacituzumab govitecan (SG) in germ cell tumor (GCT) cell lines. **A** TROP2 protein expression in a panel of GCT cell lines, including both parental and cisplatin-resistant subclones (-R) derived from embryonal carcinoma (NCCIT, 2102EP), choriocarcinoma (JAR, JEG-3, BeWo), and EC-YST intermediate (1411H), analyzed by Western blot. JEG-3, JEG-3-R, and BeWo cells show strong TROP2 expression. GAPDH served as a loading control. **B** Western blot analysis confirms a significant reduction in TROP2 protein levels in TROP2-positive cells after 72 h of treatment with 10 µg/mL SG. **C** + **D** SG-induced growth suppression is specific to TROP2-expressing GCT cell lines and their cisplatin-resistant subclones, indicating that TROP2 expression is a key factor in sensitivity to SG. Conversely, TROP2-negative cell lines are resistant to SG and do not show a notable decrease in viability. Unpaired t-test, **p < 0.01, ***p < 0.001

TROP2 protein expression is strong in parental choriocarcinoma cell lines (CC, JEG-3, BeWo) and their cisplatin-resistant subclones (e.g., JEG-3-R), while embryonal carcinoma cell lines (EC, NCCIT, 2102EP), the EC-YST intermediate line (1411H), and their resistant derivatives lack detectable TROP2 expression (Fig. 2A). This pattern aligns with the different TROP2 expression seen in metastatic GCT tissues, where CC subtypes have higher TROP2 levels compared to EC and YST subtypes (Tekin et al. 2025).

Treatment with sacituzumab govitecan (SG, 10 µg/mL for 72 h) does not increase TROP2 protein levels; instead, western blot analysis shows a significant decrease in TROP2 protein in TROP2-positive cells (JEG-3 and JEG-3-R) after SG treatment compared to untreated controls (Fig. 2B). This probably indicates SG-induced cytotoxicity and loss of viable TROP2-expressing cells, rather than direct downregulation of TROP2 (Goldenberg & Sharkey 2019; Pavone et al. 2021; Zhang et al. 2025a, b).

As shown in Figs. 2C and 2D, the cytotoxic effectiveness of SG is significantly higher in TROP2-expressing cell lines (JEG-3 and JEG-3-R) and their cisplatin-resistant subclones, demonstrated by a dose-dependent decrease in cell viability over 72 h. Meanwhile, TROP2-negative cell lines (NCCIT and NCCIT-R) resist SG, showing no notable decline in viability (unpaired t-test, *p* < 0.01). This selective toxicity aligns with the mechanism of TROP2-targeted ADCs and is backed by preclinical and clinical data from other tumor types (Goldenberg & Sharkey 2019; Pavone et al. 2021; Zhang et al. 2025a, b).

## Discussion

Cisplatin-based chemotherapy remains the standard treatment for metastatic GCTs because of its high chance of curing the disease. However, patients with cisplatin-refractory disease have limited treatment options and often poor outcomes (Cheng et al. 2018; Singla et al. 2025). Although immune checkpoint inhibitors (ICIs) have shown effectiveness in various solid tumors (Dall'Olio et al. 2022; Jacob et al. 2021), their effect in GCTs has been modest, despite high levels of PD-L1 and CTLA-4 (Lobo et al. 2019). Several clinical trials exploring ICIs, including pembrolizumab (NCT02499952), durvalumab and tremelimumab (NCT03081923), avelumab (NCT03403777), and nivolumab with or without ipilimumab (NCT03333616), have demonstrated limited benefit in this setting, probably due to the low tumor mutational burden and the lack of effective biomarker-based patient selection (Evmorfopoulos et al. 2024). Therefore, there is a clear need for new strategies such as antibody–drug conjugates (ADCs), including SG, which target tumor-specific antigens like TROP2.

TROP2 expression varies in cisplatin-refractory metastatic GCTs, with notably high TROP2 levels (H-score ≥ 200) observed in CC and TER cases. However, the therapeutic potential of TROP2-directed ADCs such as SG in TER should be approached cautiously. While TROP2 expression was moderate to strong in TER-MET(-R) and absent in normal testicular tissue, supporting its tumor specificity and potentially favorable safety profile, TER is typically a differentiated, non-proliferative component of non-seminomatous GCT and is inherently resistant to standard chemotherapy (Singla et al. 2025). The mechanism of SG, which depends on delivering a topoisomerase I inhibitor (SN-38) to actively dividing cells, is unlikely to be effective in mature TER, which lacks significant proliferative activity (Goldenberg & Sharkey 2019, 2020). This aligns with previous findings from the study by Tekin et al. (Tekin et al. 2025), showing that only a small subset of GCTs, especially CC, may be suitable for TROP2-targeted treatment.

Our in vitro findings, which demonstrate that the cytotoxic efficacy of SG correlates with TROP2 expression levels, have direct implications for the clinical usefulness of this agent in cisplatin-refractory GCTs. SG is likely to be clinically effective only in patients whose tumors show high TROP2 expression, especially in CC, where both in vivo and in vitro data show strong TROP2 positivity and drug sensitivity. Conversely, TROP2-negative GCT subtypes, such as EC and YST, are unlikely to benefit, as indicated by their resistance in cell line models.

The finding that TROP2 expression is similar in both parental and cisplatin-resistant subclones suggests that TROP2 is not a marker of cisplatin resistance itself, but rather a predictive marker for the response to TROP2-targeted therapy. Consequently, selecting patients for SG should involve directly measuring TROP2 levels in tumor tissue rather than relying solely on cisplatin resistance status (Koltai & Fliegel 2023).

Since TROP2 is absent in normal testicular tissue, the safety profile is likely to be favorable; however, the clinical benefit will probably be limited to patients with TROP2-positive tumors, especially CC and, to a lesser degree, TER (although the latter is not usually treated with systemic therapy). Routine TROP2 IHC should be considered for biomarker-driven selection of candidates for TROP2-targeted therapies, such as SG, in cisplatin-refractory GCTs (Pavone et al. 2021; Tekin et al. 2025; Zhang et al. 2025a, b).

Although our study has important strengths, several limitations must be acknowledged. First, the stability of the ADCs and off-target toxicity: SG uses a hydrolyzable linker to deliver SN-38, which can lead to premature release of the cytotoxic payload in circulation, causing systemic toxicities such as neutropenia and diarrhea, as observed in clinical trials across various solid tumors (Bardia et al. 2021a, b; Pavone et al. 2021; Zhang et al. 2025a, b). Second, the retrospective study design introduces potential biases related to patient selection, prior treatments, and tumor heterogeneity, which limit the ability to draw definitive conclusions about efficacy and safety in the broader cisplatin-refractory GCT population. Lastly, the small cohort (n = 31) reflects the rarity of cisplatin-refractory GCTs, restricting statistical power and generalizability. These factors collectively underscore the need for prospective, biomarker-driven clinical trials to confirm the safety and efficacy of TROP2-directed ADCs (e.g., SG) in this rare and heterogeneous patient population.

## Conclusion

In summary, TROP2 shows variable expression in cisplatin-resistant metastatic GCTs, with high expression most often seen in CC. SG exhibits strong cytotoxicity in TROP2-positive, chemotherapy-resistant GCT cells; however, its clinical effectiveness still needs to be confirmed.

## Supplementary Information

Below is the link to the electronic supplementary material.

Supplementary Material 1

## Data Availability

The data that support the findings of this study are available from the corresponding author upon reasonable request.

## Abbreviations

ADCs: Antibody-drug conjugates
CC: Choriocarcinoma
EC: Embryonal carcinoma
FFPE: Formalin-fixed paraffin-embedded
GCTs: Germ cell tumors
ICIs: Immune checkpoint inhibitors
IHC: Immunohistochemistry
IQR: Interquartile range
MET(-R): Cisplatin-resistant metastases
qRT-PCR: Quantitative real-time PCR (
SG: Sacituzumab govitecan
TER: Teratoma
TROP2: Trophoblast cell surface antigen 2
YST: Yolk sac tumor
