# Supplementary material for: Expression and therapeutic potential of TROP2 in cisplatin-resistant germ cell tumors
Source: J Cancer Res Clin Oncol. 2025 Oct 7;151(11):279. doi: 10.1007/s00432-025-06325-4 (PMC12504156; doi:10.1007/s00432-025-06325-4)
Supplement: Supplementary file 1 — Supplementary Material 1 [file 432_2025_6325_MOESM1_ESM.docx]

**Supporting Information – Table(s)**

Table S1. Patient characteristics of the entire germ cell tumor cohort (n = 31) and subgroups based on metastatic histology. The cohort included cases of post-chemotherapy embryonal carcinoma (EC; n = 4), choriocarcinoma (CC; n = 4), yolk sac tumor (YST; n = 14), and teratoma (TER; n = 9), treated between 2012 and 2023 at the University Hospital of Cologne.
